# Sodium thiosulfate as a potential treatment for osteoma cutis in Albright hereditary osteodystrophy: A case report

**DOI:** 10.1016/j.jdcr.2026.02.018

**Published:** 2026-02-16

**Authors:** Margaret G. Mercante, Peyton A. Yee, Shira Lanyi, Barrett J. Zlotoff

**Affiliations:** Department of Dermatology, University of Virginia, Charlottesville, Virginia

**Keywords:** Albright hereditary osteodystrophy, osteoma cutis, pediatric dermatology, sodium thiosulfate

## Introduction

Albright hereditary osteodystrophy (AHO) is a subtype of pseudohypoparathyroidism type 1a (PHP-1a) resulting from GNAS1 mutations.^1^ It is characterized by several physical features, including short stature, brachydactyly, obesity, and a round face. The phenotype also includes osteoma cutis and macular atrophic lesions representing precursors for progressive osteoma.^2^, ^3^, ^4^ Currently, surgical excision is the primary treatment option for osteoma cutis.^5^ We report a case of a pediatric patient with AHO presenting with atrophic macules representing precursors to osteoma cutis. We suggest sodium thiosulfate (STS) as a possible treatment option for osteoma cutis in AHO patients to prevent or slow the development of skin ossification.

## Case presentation

A three-year-old male with congenital hypothyroidism, PHP-1a associated with a mutation in GNAS, short stature secondary to growth hormone deficiency, hereditary alpha tryptasemia, and eczema presented to the dermatology clinic with irregular macules on the lower back, head, and legs.

At 5 months of age, the patient’s mother noticed atrophic lesions on his abdomen and multiple firm nodules on his scalp. The atrophic abdominal lesions were biopsied and were initially diagnosed as anetoderma. With the patient’s history of alpha tryptasemia, special stains for mast cells were utilized to rule this out as a cause of anetoderma. CD2 and CD25 staining showed no cutaneous mastocytosis. At 13 months, the patient underwent excisional biopsy of 3 of the firm scalp nodules. Pathology reported calcification of the lesions, consistent with osteoma cutis.

At age 17 months, the patient was referred to a pediatric geneticist due to concerns for pseudohypoparathyroidism, given the patient’s recently diagnosed osteoma cutis lesions. The patient additionally exhibited characteristic dysmorphic features including hypertelorism, epicanthal folds, short stature, and short arm length. The patient underwent whole-exome sequencing, which revealed a mutation in the GNAS gene. The patient was ultimately diagnosed with AHO. The patient was maintained on vitamin D supplementation, but given his normal levels of parathyroid hormone, phosphate, and calcium, additional treatment was not indicated.

**Fig 1:**
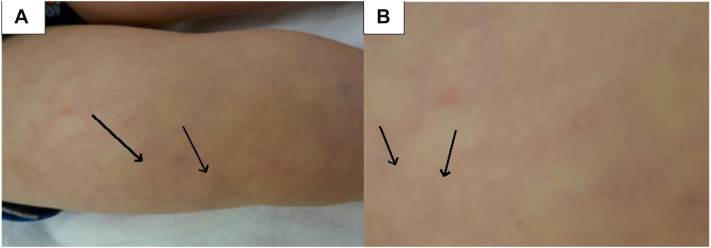
Osteoma cutis lesions characterized by arrows shown on patient’s right upper leg in an anatomical view **(A)** and enlarged **(B)**. The *arrows* are pointing to the osteoma cutis lesions.

**Fig 2:**
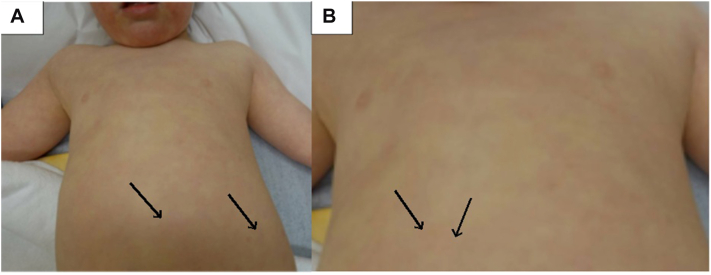
Osteoma cutis lesions characterized by arrows shown on frontal side of patient chest and stomach in an anatomical view **(A)** and magnified **(B)**. The *arrows* are pointing to the osteoma cutis lesions.

When the patient presented to the dermatology clinic at 3 years of age, multiple atrophic macules were scattered over the left abdomen, chest, bilateral lower legs, and back. Some lesions on the back had become firmer over time, with evidence of calcification (Figs 1 and 2). Given the patient’s history of AHO, the atrophic macules were suspected to represent precursor lesions of osteoma cutis rather than presumed anetoderma. The family was instructed to apply a thin layer of 25% STS cream once daily to affected areas. The 25% STS cream was obtained from a compounding pharmacy through the University of Virginia. STS treatment was well-tolerated by the patient. After a 6-month follow-up, no progression of the lesions was observed.

## Discussion

Pseudohypoparathyroidism (PHP) describes a heterogeneous group of disorders characterized by end-organ resistance to parathyroid hormone.^2^^,^^6^ AHO represents a phenotypic manifestation within the spectrum of GNAS-related disorders, which includes PHP-1a, pseudohypoparathyroidism type 1b, pseudopseudohypoparathyroidism, and progressive osseous heteroplasia.^2^^,^^6^

PHP-1a is characterized by resistance to multiple hormones, most commonly parathyroid hormone, in conjunction with the classic physical features of AHO.^2^ In contrast, Pseudohypoparathyroidism type 1b typically demonstrates isolated hormone resistance without the skeletal or cutaneous features of AHO.^2^ Pseudopseudohypoparathyroidism presents with the physical features of AHO in the absence of endocrine resistance, reflecting differences in parental inheritance of GNAS mutations.^2^^,^^6^ Progressive osseous heteroplasia represents the most severe end of the GNAS-related spectrum and is distinguished by progressive heterotopic ossification that often extends from the dermis into deeper connective tissues, leading to significant morbidity.^3^^,^^6^

Osteoma cutis, characterized by intramembranous ossification in which mesenchymal cells differentiate into bone without prior cartilage formation, is a hallmark feature of AHO.^4^ Cutaneous ossification can lead to severe disability, joint stiffness, and pain.^6^ Osteoma cutis can be the first presenting sign of AHO, especially in infants and children.^1^^,^^5^ Decreased Gsa activity due to heterozygous inactivating GNAS mutations in AHO may be associated with osteogenic differentiation in human mesenchymal cells.^1^ Besides management of pseudohypoparathyroidism, the primary treatment for osteoma cutis is surgical excision. However, excision is mainly recommended for painful lesions and aesthetic purposes, as recurrence is common.^5^

Osteoma cutis lesions are typically identified after they have already calcified, and precursor lesions are rarely described in the literature. In pediatric patients with AHO, there are some reports of atrophic skin lesions preceding osteoma cutis, as was illustrated in this case. The first report of such precursor lesions in the literature is of a five-month-old infant with PHP-1a who initially presented with linear, atrophic lesions on his trunk displaying evidence of loss of elastic fibers.^7^ However, after 6 months, all atrophic lesions developed into firm nodules that showed evidence of calcification on skin ultrasonography. Additionally, Torrelo et al recently presented a case of a newborn with AHO who had multiple atrophic skin macules on the chest and abdomen at birth, which later displayed intramembranous ossification at 1 year of age.^4^

Studies on STS as a treatment for calcinosis cutis have proposed several mechanisms, including increased calcium solubility, vasodilation, and antioxidant effect on endothelial cells, to explain its efficacy in treating calcifying lesions.^8^ It has been reported that 91.7% of pediatric patients responded to STS treatment in the setting of ectopic calcification with an excellent safety profile.^9^ For the patient presented in this case, a trial of 25% STS cream was initiated in hopes of preventing progression of the precursor lesions to osteoma cutis and improving overall quality of life. Because treatment was applied broadly rather than lesion-specifically, direct comparison between treated and untreated lesions was not possible; however, no lesions demonstrated clinical progression during the treatment interval. Reported progression from precursor atrophic lesions to clinically evident osteoma cutis ranges from several months to approximately 1 year; thus, while 6 months of stability is encouraging, longer longitudinal follow-up is necessary to determine sustained efficacy.^4^

Given the variable natural history of osteoma cutis in AHO, the optimal duration of STS therapy remains unknown. Indefinite treatment cannot be recommended based on current evidence; rather, STS may function as a temporizing or preventive therapy during periods of active lesion evolution.^9^

## Conflicts of interest

None disclosed.
